# Impact of the German Lipoprotein Apheresis Registry (DLAR) on therapeutic options to reduce increased Lp(a) levels

**DOI:** 10.1007/s11789-015-0073-1

**Published:** 2015-02-05

**Authors:** Volker J. J. Schettler, Class L. Neumann, Christian Peter, Thomas Zimmermann, Ulrich Julius, Eberhard Roeseler, Franz Heigl, The German Apheresis Working Group

**Affiliations:** 1 Centre of Nephrology Göttingen GbR, An der Lutter 24, 37075 Göttingen, Germany; 2 BRAVEBenefit for research on arterial hypertension, dyslipidemia and vascular risk and education e.V., Göttingen, Germany; 3 BioArtProducts GmbH, Rostock, Germany; 4 3rd Medical Clinic, University Hospital Carl Gustav Carus, Dresden, Germany; 5 Centre for Nephrology, Hypertension, and Metabolic Diseases, Hannover, Germany; 6 Medizinisches Versorgungszentrum Kempten Allgäu, Kempten, Germany

**Keywords:** Lipoprotein apheresis, Lipoprotein(a), Lp(a), Registry, Athersclerosis, Lipoproteinapherese, Lipoprotein(a), Lp(a), Register, Atherosklerose

## Abstract

**Background:**

The German Lipoprotein Apheresis Registry (DLAR) has been initiated by members of the Nephrology Foundation (WiNe), the German association of kidney centres (DN), the German society of nephrology (DGfN) and additional medical associations taking part in the apheresis working group. Its goal is the introduction of a substantial database, suitable to provide statistical evidence for the assessment of extracorporeal procedures. Data have been added to the DLAR since October 2011. In this article, preliminary results are first reported.

**Methods and results:**

Data are stored on a secured Internet platform. The recorded information comprises mean values and rates of change in lipid levels (cholesterol, triglyceride, low-density lipoprotein cholesterol (LDL-C), high-density lipoprotein cholesterol, lipoprotein(a) (Lp(a)) before and after apheresis therapy, blood/plasma volume, frequency and type of adverse effects, medication, vascular events, diagnoses and comorbidity. It is collected by participating apheresis centres from all over Germany. Up until October 2014, a total of 7946 lipoprotein apheresis (LA) treatments of 991 patients (787 with documented LDL-C and 688 with documented Lp(a) levels) via 96 medical accounts were documented and analysed. The current share of Lp(a) patients is 50.6 % (Lp(a) ≥ 60 mg/dl; *n* = 348/688). For both LDL-C and Lp(a), lowering rates exceeding 60 % have been observed. Likely in conjunction with these reduction rates, the preliminary analysis shows a 90 % decline in major adverse coronary events (MACE) as well as a decrease in major adverse non-coronary events (MANCE) by 69 %. As before, good tolerability and low rates of adverse effects (< 3 %) of LA therapy were found.

**Conclusions:**

The available numbers suggest in parts very good response by the participating centres to the DLAR. Unfortunately, there are also centres that have not documented any patients so far or LA treatments at all. The benchmark values for reduction rates in lipoprotein concentration required by the directives of the German Federal Joint Committee (G-BA) have all been met. The decrease in MACE and MANCE rates currently observed is very promising. However, the comparably short runtime of the registry does not allow for high confidence in the current results. Certainly, reliable data will be extractable in the coming years. Given the high interest expressed by European neighbours, the extension of the registry to the European level should be a future goal for the DLAR as well.

## Abstract (de)

**Hintergrund:**

Mitglieder der Stiftung für Nephrologie e.V. (WiNe), der Verband Deutsche Nierenzentren e. V. (DN), die Deutsche Gesellschaft für Nephrologie e. V. (DGfN) und weiterer medizinischen Fachgesellschaften der AG-Apherese riefen das Deutschen Lipoproteinapherese-Registers (DLAR) zur Schaffung einer breite Datenbasis und Evidenz der Behandlungsform ins Leben. Seit Oktober 2011 werden Daten in das DLAR eingegeben, aus dem eine erste Vorstellung der vorläufigen Ergebnisse erfolgt.

**Methoden und Ergebnisse:**

Auf einer gesicherte Internet-Plattform werden Daten zur durchschnittlichen Höhe sowie Absenkraten der Lipoproteine, behandelte Blut/Plasmavolumina, Art und Häufigkeit von Nebenwirkungen, Begleitmedikation, vaskulärer Ereignisse sowie Diagnosen und der Komorbiditäten von den teilnehmenden Apheresezentren aus Deutschland gesammelt und ausgewertet. Bis zum Oktober 2014 konnten 991 Patienten (aktuell sind bei 787 Patienten LDL-Werte und bei 688 Lp(a) –Werte dokumentiert) mit insgesamt 7946 Lipoproteinapheresen (LA) von 96 teilnehmenden ärztlichen Zugängen dokumentiert werden. Zurzeit liegt der Anteil von Lp(a)-Patienten bei 50,6 % (Lp(a) ≥ 60 mg/dl; *n* = 348/688). Sowohl für LDL-Cholesterin (LDL-C) als auch Lp(a) konnten weit mehr als 60 % -Absenkungsraten dokumentiert werden. Mutmaßlich mit diesen Absenkungsraten assoziiert zeigt sich in den ersten vorläufigen Auswertungen eine Reduktion der Major Adverse Coronary Events (MACE) um 90 % bzw. eine Major Adverse Non-Coronary Events (MANCE)-Reduktion um 69 %. Erneut konnte eine gute Verträglichkeit und geringe Nebenwirkungsrate der LA-Therapie von < 3 % dokumentiert werden.

**Schlussfolgerung:**

Aus den vorliegenden Zahlen lässt sich eine zum Teil sehr gute Resonanz der Registerteilnehmer auf das DLAR erkennen. Leider nehmen auch Zentren an dem DLAR teil, die bisher entweder keine Patienten oder keine Lipoproteinapheresebehandlungen dokumentierten. Die in den Richtlinien des Gemeinsamen Bundesausschusses (G-BA) geforderten Lipoproteinabsenkungsraten werden vollständig erfüllt. Die Datenlage der bisher erfassten MACE—und MANCE-Reduktionsraten vor und unter LA ist sehr vielversprechend, aber dennoch mit Vorsicht bei der relativ kurzen Laufzeit des DLAR zu interpretieren. Zuverlässige Aussagen lassen sich sicherlich in den kommenden Jahren treffen. Angesichts des hohen Interesses auch von europäischen Nachbarländern sollte für die Zukunft auch ein Ziel des DLAR sein, das Register auf europäische Ebene auszudehnen.

## Background

Approximately 400,000 people (ca. 5 %) in Germany may be affected by lipid disorders and sequelae [1]. In certain lipoprotein and lipid constellations, patients require lipoprotein apheresis (LA) therapy: especially in patients with severe familial hypercholesterolaemia and in cases with no response to dietary and lipid lowering therapy, but early onset or rapid progressing coronary heart disease (CHD), apoplexy or peripheral arterial disease (PAD). More than 2000 patients in Germany receive this extracorporeal treatment. This form of apheresis is designed to remove low-density lipoprotein cholesterol (LDL-C), lipoprotein(a) (Lp(a)) and other factors facilitating atherosclerosis from the blood. Since 1974, six different methods for LA have been developed. All LA techniques approved in Germany meet the quality standards required by the German Federal Joint Committee (G-BA) in that they achieve a minimum 60 % reduction of LDL-C and Lp(a) concentrations in a single treatment [2]. The extracorporeal therapy is received weekly in most cases as an ambulant treatment in the dialysis facility of a resident nephrologist, in specialised hospitals or university clinics as well as in respective charities.

Members of the Nephrology Foundation (WiNe), the German association of kidney centres (DN), the German society of nephrology (DGfN) and additional medical associations taking part in the apheresis working group (The German Society for Combating Dyslipidemia and its Associated Illnesses (DGFF e. V.—Lipid-League), the German Cardiac Society (DGK), the German Society for Clinical Chemistry and Laboratory Medicine e. V. (DGKL), the International Society for Apheresis) initiated the first national German Lipoprotein Apheresis Registry (DLAR). All technical requirements, input forms and database systems were implemented by BioArtProducts GmbH. The responsible Nephrology Foundation has commissioned its Wissenschaftliches Institut für Nephrologie (WiNe—Scientific Institute for Nephrology) as operator of the DLAR. With WiNe in this position, German data protection laws are met and funding has been secured until 2019.

In addition to the scientific interest, the initiative has been motivated to meet the demand of the G-BA for systematic investigation and analysis of the permanent impact of LA therapy with regard to the occurrence of additional cardiovascular events and, if applicable, associated mortality. It aims to validate and thus secure LA methods as established lipid-lowering therapy and to justify the comparably high costs of therapy for a relatively low number of LA patients. An originally required randomised, prospective study on the extracorporeal removal of Lp(a), (The ELAILa trial) involving a control group not undergoing LA was refused by the ethical review committee of the Berlin Charité, Germany.

In a pilot phase starting October 2011, data were collected by seven larger apheresis centres (AC). The experiences gathered here were employed to optimise the data entry process of the registry towards the version currently in use. Since the end of the pilot phase in March 2012, limited funding currently fixes the project runtime from April 2012 to December 2016, which is actually prolonged to June 2019. Participation in the registry is voluntary and free of charge for the participants.

The users of the registry contribute to add a large sample to the hitherto positive results of single observations and existing publications. Furthermore, they receive higher quality data on patient population, morbidity, reduction rates, treatment volumes, accompanying medication and compatibility [3, 4]. The registries meet the requirements of the G-BA for a systematic survey of the LA therapy, technically equating to quality assurance for the method as well. Also, the documented data are suitable to inform the annual follow-up applications on continuation of LA therapy to the apheresis commission of the respective federal states. However, this mode of application is currently only officially recognised by the Regional Associations of Statutory Health Insurance Physicians of Lower Saxony (KVN).

This article presents results of a first, preliminary evaluation of data collected by the DLAR.

## Methods and results

### Description of the registry

The DLAR is a secure Internet platform providing participating centres with an input mask for data on treatments observing all data law requirements. Here, the following variables are recorded: Mean concentration of lipid levels (cholesterol, triglyceride, LDL cholesterol (LDL-C), high-density lipoprotein cholesterol, Lp(a)), mean reduction rates of lipids before and after apheresis, mean treated volume of blood/plasma, type and frequency of adverse effects and complication, accompanying medication, type and number of vascular events involving the heart (Major adverse coronary events (MACE)) or other organs (Major adverse non-coronary events (MANCE)) observed during therapy in comparison with anamnestic data, lipidological diagnosis and comorbidities as well as a description of vascular accesses. The scientific advisory board of the DLAR has requested at least one complete LA treatment to be recorded every 3 months. All new events (MACE, MANCE) occurring under LA therapy have to be documented appropriately in the registry. Retracing single patients from the dataset is not possible to the funding body, the operator or the service providers. Each participating centre has full access to its data, which are decoded client-side.

The foundation owned Wissenschaftliches Institut für Nephrologie (WiNe—Scientific Institute for Nephrology, Düsseldorf, Germany) was commissioned as operator of the DLAR. The Stiftung für Nephrologie (Nephrology Foundation) is as funding body of the DLAR responsible for financing, incorporating project-bound donations of the industrial partners involved (B. Braun Avitum GmbH, DIAMED Medizintechnik, Fresenius Medical Care Deutschland GmbH, Kaneka Pharma Europe N.V., Miltenyi Biotec GmbH; Germany) as well. Therefore, no costs are created for the participating centres. The company BioArtProducts GmbH (B.A.P., Rostock, Germany) was commissioned with the technical implementation of the registry as well as the preliminary statistical data analysis. In assessing data quality and analysis, WiNe is supported by a scientific advisory board, the members of which are listed in the acknowledgements of this publication.

### Results

Seven LA centres were selected by the scientific advisory board to participate in the pilot phase (Start: 1 October 2011 to End: 15 May 2012) of the DLAR. Five of the centres subsequently entered data into the registry. For 301 apheresis patients, complete data on 455 LA treatments were gathered. Adverse events were recorded for 14 treatments (3 %) (failed punctures *n* = 2, hypotension *n* = 2, nausea *n* = 1, abdominal pain *n* = 1, technical difficulties *n* = 1, other *n* = 6). For the patients, 301 diagnosis related groups were entered (CHD *n* = 280, heart transplantation *n* = 5, PAD *n* = 13, cerebrovascular diseases *n* = 3).

**Fig. 1:**
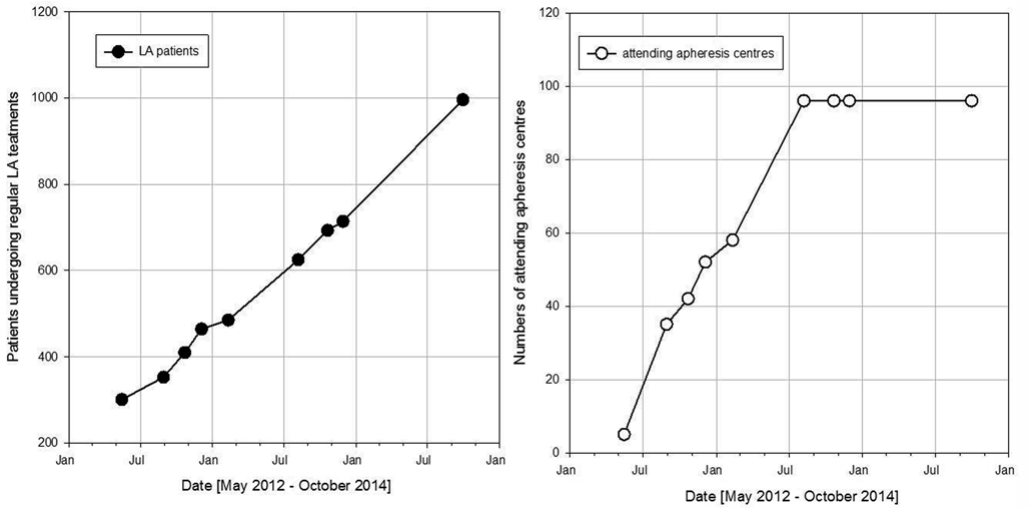
Number of documented patients (*left*) and participating centres (*right*) over the time period from May 2012 to October 2014

**Fig. 2:**
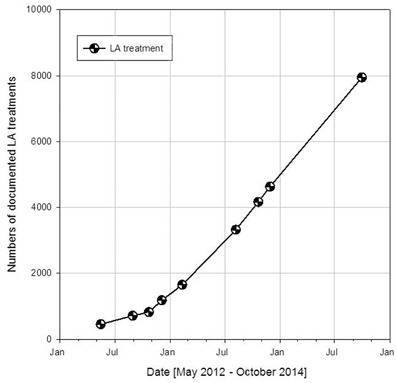
Number of documented lipoprotein apheresis treatments over the time period from May 2012 to October 2014

From the start of the registry in May 2012 until October 2014, the number of registered patients receiving chronic LA treatment increased continuously from 301 (pilot phase) to 991 patients in 96 participating AC (Fig. 1). Of those patients included into the DLAR, the diagnosis prior to the initialisation of LA therapy is known for 887 only. Of these, 630 suffered from CHD, 111 from a cerebrovascular insult and 146 from PAD. This also led to a distinct increase in documented LA treatments to a total of 7946 LA therapies (Table 1, Fig. 2).

**Table 1:** Breakdown of patients and treatments per centre

	October 2014	June 2014	2013	2012
Participating centres	96	96	96	96
Total collected patients	991	918	873	680
100+ Patients	3	1	1	1
50–99 Patients	3	5	4	3
10–49 Patients	16	14	14	10
1–9 Patients	38	38	36	
0 Patient	36	38	41	
Collected treatment	7946	6535	3158	3752
Centres with 100+ treatments	14	13	12	
Centres with 10–99 treatments	18	16	17	
Centres with 0–9 treatments	64	67	67	

The currently participating 96 AC are comprising centres, which attend to more than 100 LA patients as well as less than 10 (Table 1). Here, it is remarkable that the data recorded in the DLAR are mainly provided by the largest AC. It is further to note that there are AC that have registered with the DLAR but have not entered any patients so far. Furthermore, some AC have entered patient data but not documented any LA treatments.

Of the 991 patients in the register, data recorded prior to initiation of LA therapy have been recorded of LDL-C concentration for 787 patients and Lp(a) concentrations for 688 patients, amounting to approximately 10 % of laboratory data missing. Patients can be divided in the following subgroups: 400 patients with LDL-C > 3.4 mmol/l (130 mg/dl) (50.8 %) and 387 patients with LDL-C < 3.4 mmol/l (130 mg/dl) (49.2 %), respectively and 539 patients with LDL-C > 2.6 mmol/l (100 mg/dl) (68.5 %) and 248 patients with LDL-C < 2.6 mmol/l (100 mg/dl) (31.5 %) (*n* = 787), respectively and 348 patients with Lp(a) > 60 mg/dl (50.6 %) (*n* = 688).

This Lp(a)-subgroup (*n* = 348) is divided into 86 patients with Lp(a) > 60 mg/dl and LDL-C > 3.4 mmol/l (130 mg/dl) (24.7 %) and 262 patients with Lp(a) > 60 mg/dl and LDL-C < 3.4 mmol/l (130 mg/dl) (75.3 %), respectively, and 172 patients with Lp(a) > 60 mg/dl and LDL-C > 2.6 mmol/l (100 mg/dl) (49.4 %), respectively, and 176 patients with Lp(a) > 60 mg/dl and LDL-C < 2.6 mmol/l (100 mg/dl) (50.6 %).

**Fig. 3:**
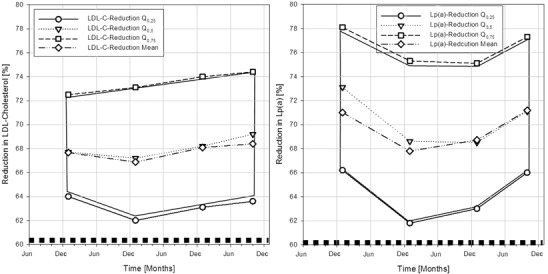
Reduction rates of low-density lipoprotein cholesterol (*left*) and lipoprotein(a) (*right*) under chronic lipoprotein apheresis treatment over the time period from December 2011 to October 2014

**Fig. 4:**
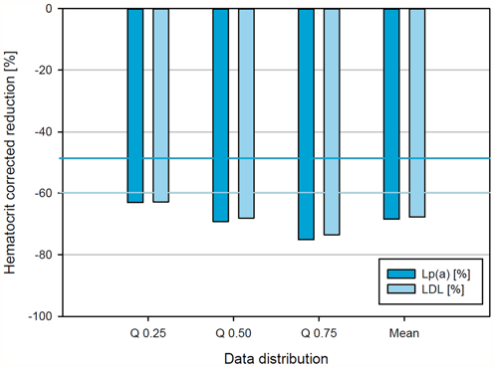
Haematocrit-corrected reduction of S-LDL + S-Lp(a) concentrations in percentage after lipoprotein apheresis over the time period from December 2011 to October 2014; data distributions are given as quartiles and mean

Reduction rates of LDL-C and Lp(a) under chronic LA treatment were recorded in the DLAR (Fig. 3). Furthermore, existing data allowed the creation of a sample taking in addition the haematocrit-corrected LDL-C and Lp(a) reduction into account (Fig. 4—online). All LA recorded showed a reduction of the lipoproteins LDL-C and Lp(a) by more than 60 %. A pronounced haemodilution effect seems to be absent.

For the time period from 2011 to 2014, preliminary analysis finds a reduction of MACE by 90 % as well as a reduction in MANCE by 69 % for LA patients in the registry.

Over the course of the year 2013, adversary effects were recorded for 88 (2.93 %) patients, where in 70 (2.33 %) cases exactly one effect was reported and in 18 (0.6 %) more than one. During 2012, the total was 49 (2.98 %), 36 (2.19 %) of which with one adversary effect and 13 (0.79 %) with more than one. It is remarkable that the most reported adversary effects were failed punctures and no direct side effects of the LA therapy were documented.

## Discussion

The constant increase in the numbers of participating AC and documented patients since the start of the DLAR allows to infer positive reception and high motivation of practitioners and patients alike. Still there exist AC that do not record patient data and/or LA treatments. A hearing found different reasons stated by the respondents, e.g. too little time for documentation, lack of manpower or not enough patients. Presumably, more than 2000 patients in Germany undergo regular LA therapy. Therefore, WiNe will continue to promote the voluntary participation of AC in the DLAR with the goal to register at least 50 % of German LA patients in the registry.

The preliminary extended data analysis showed that 50.6 % of all patients had available laboratory data (*n* = 688) and had a raised Lp(a) value of which 25.6 % (*n* = 176) showed isolated Lp(a) elevation (Lp(a) > 60 mg/dl, LDL-C < 2.6 mmol/l (100 mg/dl)) of all data. The share of Lp(a) patients undergoing chronic LA therapy was significantly lower in earlier studies [5]. This change in share of Lp(a) patients can in parts be explained by the use of newer, more effective statins like Atorvastatin and Rosuvastatin, which made LA therapy unnecessary for many patients already before the start of the DLAR. Furthermore, the assessment of cardiovascular risk factors increasingly includes tests for Lp(a) concentrations, since Lp(a) was established as independent cardiovascular risk factor [6]. Due to the negative results of the AIM-HIGH trial, nicotinic acid has been withdrawn from the German market, leaving LA therapy at the moment as only means to potentially achieve Lp(a) reduction. A new therapy approach is currently being investigated (in part already in phase 3 studies): the PCSK9 inhibition [7]. The currently farthest developed PCSK9 antibodies do achieve next to a considerable LDL-C reduction a moderate decrease by approximately 30 % in Lp(a) as well [8]. Whether these reductions do influence cardiovascular events or mortality needs to be confirmed by current studies. With respect to additional, efficient Lp(a) reduction, PCKS9 inhibition may develop into an add-on therapy to LA [9, 10].

Earlier LA studies have established that after initiation of LA therapy, the recurrence of cardiovascular events MACE and MANCE can be reduced significantly, especially if Lp(a) is reduced aggressively [9, 11]. The preliminary data analysis now shows already a significant MACE and MANCE reduction by 90 and 69 %, respectively in patients undergoing chronic LA treatments in agreement with earlier observations [12]. Due to the relatively short runtime of the registry and the current data availability, these results have to be treated cautiously. In this light, an extension of the project life beyond the projected year 2016 seems expedient to further increase the significance of data derived from the DLAR, which is actually prolonged to June 2019. An extension of the register to the European level could contribute to an increase in data quantity and quality as well. At the moment however this project lack financial support.

## Conclusion

The DLAR represents an important means to meet requirements for quality assurance of LA therapy expressed both by the G-BA as well as users. Already now the existing data document the high quality of LA treatment and thus could justify the introduction of a nationally standardised rate of refund for LA in Germany by the National Association of Statutory Health Insurance Physicians.

## Abbreviations

DLAR: German Lipoprotein Apheresis Registry
Lp(a): Lipoprotein(a)
DN: Verband Deutsche Nierenzentren e. V. (German association of kidney centres)
DGfN: German Society of Nephrology
WiNe: Scientific Institute for Nephrology
MACE: Major adverse coronary events
MANCE: Major adverse non-coronary events
G-BA: German Federal Joint Committee
KBV: National Association of Statutory Health Insurance Physicians
